# MicroRNA-210 Regulates Endoplasmic Reticulum Stress and Apoptosis in Porcine Embryos

**DOI:** 10.3390/ani11010221

**Published:** 2021-01-18

**Authors:** Muhammad Rosyid Ridlo, Eui Hyun Kim, Geon A. Kim

**Affiliations:** 1 Department of Theriogenology and Biotechnology, Research Institute for Veterinary Science, College of Veterinary Medicine, Seoul National University, Seoul 08826, Korea; rosyidridlodrh@gmail.com (M.R.R.); hyun9214@snu.ac.kr (E.H.K.); 2 Department of Bioresources Technology and Veterinary, Vocational College, Universitas Gadjah Mada, Yogyakarta 55281, Indonesia; 3 Department of Biomedical Laboratory Science, School of Medicine, Eulji University, Daejon 34824, Korea

**Keywords:** miR-210-inhibitor, miR-210-mimic, endoplasmic reticulum stress, apoptosis, in vitro culture, parthenogenetic activation, pig

## Abstract

**Simple Summary:**

The purpose of this study was to explore the effect of miR-210 on in vitro embryo development, mRNA expression related endoplasmic reticulum (ER) stress. Treatment with a miR-210-inhibitor significantly improved in vitro embryo development and total blastocyst cell number (TCN). Furthermore, miR-210-inhibitor treatment downregulated ER stress and apoptosis-related gene expression, while simultaneously improving embryo capacity. In contrast, a miR-210-mimic decreased in vitro embryo development, TCN, upregulated ER stress and apoptosis genes, and concomitantly impaired embryo quality. Therefore, we suggest that miR-210 plays an important role in porcine in vitro embryo development.

**Abstract:**

Endoplasmic reticulum (ER) stress can be triggered during in vitro embryo production and is a major obstacle to embryo survival. MicroRNA (miR)-210 is associated with cellular adaptation to cellular stress and inflammation. An experiment was conducted to understand the effects of miR-210 on in vitro embryo development, ER stress, and apoptosis; to achieve this, miR-210 was microinjected into parthenogenetically activated embryos. Our results revealed that miR-210 inhibition significantly enhanced the cleavage rate, blastocyst formation rate, and total cell number (TCN) of blastocysts, and reduced expression levels of *XBP1* (*p* < 0.05). miR-210 inhibition greatly reduced the expression of ER stress-related genes (*uXBP1*, *sXBP1*, *ATF4*, and *PTPN1*) and *Caspase 3* and increased the levels of *NANOG* and *SOX2* (*p* < 0.05). A miR-210-mimic significantly decreased the cleavage, blastocyst rate, TCN, and expression levels of *XBP1* compared with other groups (*p* < 0.05). The miR-210-mimic impaired the expression levels of *uXBP1*, *sXBP1*, *ATF4*, *PTPN1*, and *Caspase 3* and decreased the expression of *NANOG* and *SOX2* (*p* < 0.05). In conclusion, miR-210 plays an essential role in porcine in vitro embryo development. Therefore, we suggest that miR-210 inhibition could alleviate ER stress and reduce apoptosis to support the enhancement of in vitro embryo production.

## 1. Introduction

MicroRNAs (miRNAs) are small noncoding RNAs, 18–25 nucleotides in length, that post-transcriptionally regulate their target messenger RNAs (mRNAs), usually by targeting the 3′ untranslated regions (3′ UTRs) or noncoding regions of these target mRNAs [1,2]. Expression of miRNAs during germinal vesicle (GV) to metaphase II oocytes (MII) stage was investigated in porcine oocytes. Expression of miR-210 and miR-27b-3p was significantly lower in MII oocytes compared with those from GV oocytes, and investigation of miR-2, miR-10a-5p, miR-486, miR-10b, and miR-183 revealed higher expression in the MII stage, and an estimated fold change >2 in GV and MII oocytes [3]. Localization of miRNAs has been detected in eight-cell embryos, MII, and blastocysts stage in porcine. Investigation of miR-16, -21, -23b, -205, and -195 revealed that they were highly expressed in MII. Expression of miR-17, -125b, -125a-5p, -128, and -205 was highly detected in eight-cell embryos, and detection of miR-92a, -129-5p, -205, -210, and -302a was most highly expressed in blastocyst [4]. Moreover, the highest ranked of the most expressed miRNAs in blastocyst were hsa-miR-200a-3p, sscmiR-210, bta-miR-21-5p, bta-miR-1246, and bta-miR-378d, ranging from ∼6000 to ∼62,000 read counts [5].

miRNAs play a role in gene expression by influencing the translation of mRNA target genes, and some miRNAs are expressed interdependently of their target mRNA to promote degradation [6]. Abnormal expression of miRNAs can drastically change the translation of some genes, thereby influencing the phenotype of cells [7]. Micro-RNA editing may result in the targeting of various mRNAs, thus influencing the functions of RNA-mediated gene complexes [8].

Reduction or an increase in miRNAs has been associated with various clinical diseases, ranging from malignancy to myocardial localized necrosis [9], sickle cell infection, endometrium disease, lung, liver, kidney disease [10], immune system diseases of skin, and psychiatric and neurodegenerative diseases [11]. Many studies have revealed that microRNA (miR)-210 plays an important role in many pathologies and diseases, for example, in the cellular response to hypoxia, which influences cell survival and differentiation [12]. The infusion of double-stranded miR-210 has also been shown to improve recuperation of the partially torn anterior cruciate ligament (ACL) by improving angiogenesis via upregulation of fibroblast growth factor 2 (FGF2) and vascular endothelial growth factor (VEGF) [13]. A previous study reported that miR-210 is increased in the majority of tumors and is associated with poor clinical results [14]. In neural stem cells (NSCs), miR-210 inhibition improved mitochondrial oxidative metabolism. Inhibition of miR-210 during inflammation effectively protects mitochondria and improves the activities of cytochrome C oxidase and aconitase [15]. Transfection with a miR-210 inhibitor suppresses cell migration and invasion of human osteosarcoma cell lines and in osteosarcoma mice [16]. Furthermore, a study in a neonatal rat hypoxic-ischemic encephalopathy (HIE) model revealed that inhibition of miR-210 provided a neuroprotective result [17].

Studies on miRNAs have progressively suggested that various miRNAs are involved in diverse biological processes, including pregnancy [18], implantation [19], zygotic genome activation (ZGA), early embryonal development [20], fertilization, and gametogenesis [21,22]. In addition, parental miRNAs and endometrial miRNAs from the uterine fluid could be involved in maternal-embryo interactions and play a significant role by influencing the expression of genes related to embryonic development [23,24]. MiRNAs exert significant effects during the development of zygotes to pluripotent blastocysts and the progression of fertilized oocytes to pluripotent blastocysts [20,25,26]. Nonetheless, most miRNAs demonstrate a fluctuating expression according to the phase of embryonic development. Furthermore, some miRNAs are phase-specific [27]. In light of these reports, parental miRNAs have been hypothesized to play an essential, yet restricted role during fertilization and in ZGA; furthermore, miRNAs may play an important role during the developmental progression from ZGA to the pluripotent blastocyst [23]. Previous studies have reported that miR-210 is related to the modulation of cellular stress [28,29]. MiR-210 impairs mitochondrial function, increases glycolysis, and triggers the generation of reactive oxygen species (ROS) [29,30]. Overexpression of miR-210 was associated with colorectal cancer and could activate cancer cell apoptosis [29,31]. One study related to glioblastoma reported that there was a close relationship between miR-210 and ER stress; this finding provides a new perspective on the utilization of miRNA interference for future study [32].

Thus far, there have been no studies on the connection between miR-210 and in vitro embryo development and consecutive endoplasmic reticulum (ER) stress. Therefore, in this experiment, we explored the impact of miR-210 applications (inhibition and mimic) on cleavage, blastocyst rate, and gene expression levels of mRNAs related to ER stress, apoptosis, and embryo quality.

## 2. Materials and Methods

### 2.1. Research Ethics and Chemicals

Screening of the experimental ethics regarding the utilization of ovaries was completed according to the Institutional Animal Care and Use Committee (IACUC) of Seoul National University (approval no. SNU-190621-2). All chemical compounds used in this experiment were obtained from Sigma-Aldrich Chemical Company (St. Louis, MO, USA), unless otherwise stated.

### 2.2. Retrieval of Oocyte and In Vitro Maturation (IVM)

The ovaries of prepubertal gilts were collected from a local abattoir and transported to the laboratory at 32–37 °C. Cumulus–oocyte complexes (COCs) were sliced using sterilized forceps and blades. The COCs were then washed three times in washing medium containing 9.5 g/L of tissue culture medium-199 (1x) Earle’s salts (Cat. No. 31100-027) (Thermo Fisher Scientific, MA, USA), 5 mM sodium hydroxide, 10 mM *N*-piperazine-*N*’-[2-ethanesufonic acid] (HEPES), 0.3% polyvinyl alcohol (PVA), 2 mM sodium bicarbonate, and 1% penicillin-streptomycin (Invitrogen). The COCs with ≥3 layers of cumulus cells (CCs) and a dark homogenous cytoplasm were selected for the experiment. The selected immature oocytes were cultured in IVM medium consisting of tissue culture medium-199 (1x) Earle’s salts (Cat. No. 11150-059), 10 μL/mL insulin-transferrin-selenium solution (ITS-A) 100x (Invitrogen), 10 IU/mL equine chorionic gonadotropin (eCG), 10 IU/mL human chorionic gonadotropin (hCG), 10 ng/mL epidermal growth factor, 0.91 mM sodium pyruvate, 0.57 mM cysteine, and 10% porcine follicular fluid (*vol/vol*). The COCs were cultured at 39 °C, 5% CO_2_ in 95% humidified air. After 22 h of in vitro maturation culture with hormones, the COCs were rinsed with fresh hormone-free IVM medium and then incubated in hormone-free IVM medium for a further 22 h.

### 2.3. Electrical Activation of Porcine Oocytes

Cumulus–oocyte complexes (COCs) were denuded by pipetting in 0.1% hyaluronidase. Denuded oocytes were equilibrated in pulsing medium consisting of 0.28 M mannitol, 0.1 mM CaCl_2_, 0.5 mM HEPES, and 0.1 mM MgSO_4_, and then transferred into a glass chamber containing two electrodes overlaid with the pulsing medium connected to a BTX Electro-Cell Manipulator 2001 (BTX Inc., San Diego, CA, USA). Oocytes were activated with a single direct current (DC) pulse of 1.5 kV/cm for 60 μs. The oocytes were then washed and transferred to porcine zygote medium-5 (PZM-5) (Waco Chemicals, Osaka, Japan, Cat. # CSR-CK024), and then cultured at 39 °C in a humidified atmosphere of 5% O_2_, 5% CO_2_, and 90% N_2_.

### 2.4. Microinjection of Porcine Oocytes

Before microinjection, approximately 40 activated oocytes were placed in a 4 μL droplet of culture medium under oil. Oocytes were activated by parthenogenetic activation using an electrical activation machine (BTX Inc., San Diego, CA, USA). The handling and manipulation of zygotes were performed under an inverted microscope (Eclipse TE2000-S; Nikon Imaging Japan, Tokyo, Japan). Microinjection was performed using the methods described in previous reports with some modifications [33,34,35,36]. In brief, microinjection was performed in zygotes 6 h after parthenogenetic activation using a sterile injection capillary (Femtotip II; Eppendorf, Hamburg, Germany) connected to the Femtojet system (Eppendorf, Hamburg, Germany). We utilized an artificial synthetic cfa-miR-210-inhibitor (Cat. R-200121-0109. Bioneer, Daejeon, Korea) and a cfa-miR-210-mimic (Cat. *p*-200121-0109. Bioneer, Daejeon, Korea); the list of micro-RNAs used is presented in Table 1. MiR-210 was microinjected into the cytoplasm of embryos at a concentration of 20 pmol/μL [35]. Injection success was confirmed by visualization of an injected droplet (10 pL per injection) and movement within the cytoplasm of the zygote. Microinjected embryos were subsequently rinsed and cultured in PZM-5 according to the distribution of the group.

### 2.5. Embryo Development and Total Cells Blastocyst Number after Activation

Evaluations of cleavage and blastocyst development were performed on days 2 (48 h) and 7 (168 h), respectively. The total cell number (TCN) count was performed on day 7 (168 h). Blastocysts were rinsed using TALP medium. Bisbenzimide (Hoechst-33342) 5 μg/mL was utilized for nuclear staining for 10 min in a dark environment. Afterwards, blastocysts were rinsed and placed in a glycerol drop on a glass slide, which was then gently covered with a microscope cover glass. Observations were performed using an inverted microscope equipped with epifluorescence (Nikon Corp, Tokyo, Japan) at 400× magnification. Images were analyzed using Image J software (version 1.49 q; National Institutes of Health, Bethesda, MD, USA).

### 2.6. The X-Box Binding Protein 1 (XBP1) Immunofluorescence Staining in Blastocyst

After 7 days of embryo culture, blastocysts were rinsed three times with phosphate buffered saline (PBS) containing 1% polyvinyl alcohol. Then, blastocysts were fixed in 4% paraformaldehyde (*w/v*) in PBS for 1 h at room temperature. After fixation, blastocysts were transferred to distilled water (DW) containing 1% (*v/v*) Triton X-100 for 1 h at 38 °C. Blastocysts were then blocked to prevent non-specific binding for 2 h in PBS with 2% bovine serum albumin (BSA) at 38 °C. Embryos were then incubated with *XBP1* primary antibody diluted in 2% BSA in PBS (1:400; PA5-27650; Invitrogen, Carlsbad, CA, USA) at 4 °C overnight. Then, secondary fluorescein isothiocyanate-conjugated anti-rabbit polyclonal antibody diluted in 2% BSA in PBS (1:200; ab6717; Abcam, Cambridge, UK) was utilized at 25 °C for 2 h in the dark. After immunofluorescence staining of *XBP1* was completed, counterstaining was performed with 5 μg/mL Hoechst-33342 for 10 min. Blastocysts were mounted on glass slides, flattened softly with cover glass, and evaluated with a fluorescence microscope (Nikon Corp., Tokyo, Japan). Next, the intensities of *XBP1* (green) were evaluated by analyzing the sample images with ImageJ software (version 1.49 q; National Institute of Health, Bethesda, MD, USA).

### 2.7. Analysis of Gene Expression in Blastocysts by Quantitative Real-Time Polymerase Chain Reaction (qRT-PCR)

The blastocyst samples were collected, washed with PBS, and stored at −80 °C until use. At least 70 blastocysts from each group were utilized for RNA extraction with the RNAqueousTM Micro Kit (Invitrogen, Vilnius, Lithuania). A NanoDrop 2000 Spectrophotometer (Thermo Fisher Scientific, Wilmington, DE, USA) was used for mRNA quantification. As indicated by the manufacturer’s protocols, complementary DNA (cDNA) synthesis was implemented using the amfiRivert cDNA synthesis Platinum Master Mix 0 (GenDEPOT, Houston, TX, USA). The qRT-PCR protocol was explained in a previous report [37]. In brief, mixtures of each reaction containing 0.4 μL (10 pmol/mL) reverse primer, 0.4 μL (10 pmol/μL) forward primer, 8.2 μL of nuclease free water (NFW), 10 μL SYBR Premix Ex Taq (Takara, Otsu, Japan), and 1 μL of cDNA were added to a PCR plate (Micro-Amp Optical 96-Well Reaction Plate, Applied Biosystems, Singapore) according to the experimental design. Amplification was performed using the StepOneTM Real-Time PCR System (Applied Biosystems, Waltham, MA, USA) in a thermal cycler. Up to forty reaction cycles were performed with the protocol: denaturation at 95 °C for 15 s, annealing at 60 °C for 1 min, and extension at 72 °C for 1 min. At least three biological replicates and four technical replicates were used for each plate. The mRNA levels of target genes were normalized to the endogenous control gene glyceraldehyde-3-phosphate dehydrogenase (*GAPDH*). The relative expression of genes was analyzed by applying the equation R = 2^−^ [ΔCt sample − ΔCt control]. The list of primer sequences is shown in Table 2.

### 2.8. Research Outline

This study aimed to elucidate the effects of an miR-210 inhibitor and mimic on porcine embryos using microinjection. In the first experiment, we designed three experimental groups: (i) control (injected with diethylpyrocarbonate (DEPC) water); (ii) miR-210-inhibitor; and (iii) miR-210-mimic. We investigated the cleavage rate, consecutive in vitro embryo development progress, and total blastocyst cell numbers. In the subsequent analysis, we evaluated the expression levels of *XBP1* using immunofluorescence staining. In the third experiment, we analyzed expression levels of mRNAs related to unfolded protein response (UPR)-related genes, *Caspase 3*, *NANOG*, and *SOX2* in the blastocyst stage.

### 2.9. Statistical Analysis

All data were analyzed using GraphPad PRISM ver.5.01 (PRISM 5; GraphPad Software, Inc., San Diego, CA, USA). Data from the first experiment were analyzed using univariate analysis of variance (ANOVA) followed by Tukey’s test. Data from the second and third experiments concerning gene expression levels were performed with Student’s *t*-test. Differences were considered statistically significant at *p* < 0.05.

## 3. Results

### 3.1. Effects of miR-210 (Inhibitor and Mimic) Injection on Cleavage, Blastocyst Rate, and Total Blastocyst Cell Number

We observed the effects of microinjection of miR-210 (inhibitor and mimic) on in vitro embryo development in the first experiment. The cleavage rate, blastocyst formation rate, and TCN results are presented in Table 3. Cleavage rate development of embryos was significantly enhanced in the miR-210-inhibitor group (*p* < 0.05). This result was the highest rate of cleavage compared with the other groups. Treatment with the miR-210-mimic showed significantly reduced embryo cleavage rates compared with the control and miR-210-inhibitor group (*p* < 0.05). Consecutive in vitro embryo development on blastocyst formation rates revealed a similar pattern to cleavage development among the groups. The miR-210-inhibitor group yielded the highest rate of blastocyst formation, followed by the control group, and the miR-210-mimic group had the significantly lowest rate of blastocyst formation (*p* < 0.05). The TCN was significantly increased in the miR-210-inhibitor group compared with the control and miR-210-mimic groups (*p* < 0.05). The highest TCN was the miR-210-inhibitor group, followed by the miR-210-mimic and control groups, respectively.

### 3.2. Expression Levels of XBP1 in Embryos after Microinjection of miR-210 (Inhibitor and Mimic)

**Figure 1:**
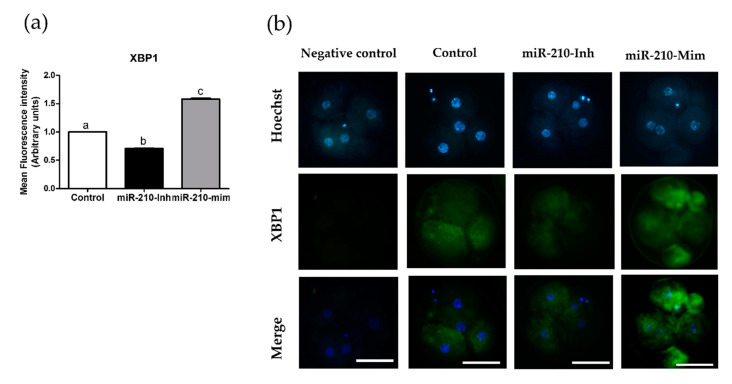
Expression levels of *XBP1*, shown as fluorescence intensity at cleavage stage of microinjected embryos. (**a**) Chart showing the assessment of fluorescence intensity of *XBP1*. At least 24 embryos per group from four biological replicates were analyzed. Data are presented as mean ± SEM. Treatment groups pointed out with letters are considered to be statistically significant (*p* < 0.05). (**b**) Images showing immunofluorescence staining of embryos (green) in the control, miR-210-inhibitor, and miR-210-mimic treatment groups. Hoechst staining was used to stain DNA (blue), and merged images were created to demonstrate colocalization (scale bars 50 μm; 400× magnification). miR-210-Inh, micro-RNA-inhibitor; miR-210-Mim, micro-RNA-mimic; SEM, standard error of mean.

**Figure 2:**
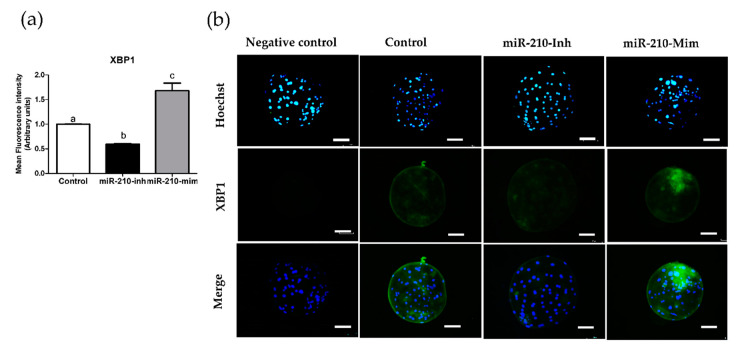
Expression levels of *XBP1*, shown as fluorescence intensity in blastocysts of microinjected embryos. (**a**) Chart showing the assessment of fluorescence intensity of *XBP1*. At least 20 embryos per group from four biological replicates were analyzed. Data are presented as mean ± SEM. Treatment groups pointed out with letters are considered to be statistically significant (*p* < 0.05). (**b**) Images showing immunofluorescence staining of blastocysts (green) in the control, miR-210-inhibitor, and miR-210-mimic treatment groups. Hoechst staining was used to stain DNA (blue), and merged images were created to demonstrate colocalization (scale bars 50 μm; 400× magnification). miR-210-Inh, micro-RNA-inhibitor; miR-210-Mim, micro-RNA-mimic; SEM, standard error of mean.

In the second experiment, we analyzed the expression levels of *XBP1* at the cleavage (Figure 1) and blastocyst (Figure 2) stages resulting from miR-210 inhibitor and mimic treatment. Expression of *XBP1* was significantly reduced in the miR-210-inhibitor group compared with the other groups (*p* < 0.05). The miR-210-mimic significantly increased the protein expression levels of *XBP1* compared with the miR-210-inhibitor and control group (*p* < 0.05).

### 3.3. Effects of miR-210 (Inhibitor and Mimic) Treatment on Endoplasmic Reticulum Stress-Related Genes, Caspase 3, NANOG, and SOX 2 in Blastocysts

**Figure 3:**
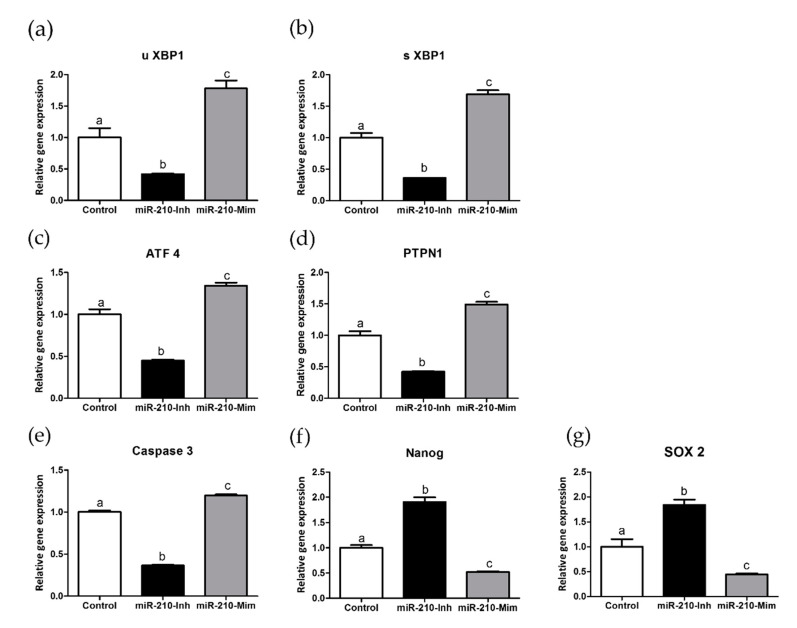
Gene expression levels of *uXBP1*, *sXBP1*, *ATF4*, *PTPN1*, *Caspase 3*, *NANOG*, and *SOX2* (**a**–**g**) in microinjected blastocysts. Data information is presented as mean ± SEM. Treatment groups pointed out with letters are considered to be statistically significant (*p* < 0.05). At least 70 blastocysts were used in each group, with technical replications repeated in triplicates for the real-time PCR analysis. miR-210-Inh, micro-RNA-inhibitor; miR-210-Mim, micro-RNA-mimic; SEM, standard error of mean.

We evaluated the effects of miR-210 inhibitor and mimic treatment on ER stress-related genes, such as activating transcription factor-4 (*ATF4*), protein tyrosine phosphatase non-receptor type 1 (*PTPN1*), unspliced *XBP1* (*uXBP1*), spliced *XBP1* (*sXBP1*), the apoptosis-related gene, *Caspase 3*, and genes related to embryo pluripotency such as *NANOG* and *SOX 2*. The analysis results of the molecular work are presented in Figure 3. In the presented study, miR-210-inhibitor treatment significantly downregulated the expression levels of UPR-related genes (*uXBP1*, *sXBP1*, activating transcription factor-4 (*ATF4*), and protein tyrosine phosphatase non-receptor type 1 (*PTPN1*) compared to the control (*p* < 0.05). On the other hand, miR-210-mimic treatment significantly upregulated the UPR-related genes compared with the control group (*p* < 0.05). In apoptosis-related gene analysis, miR-210-inhibitor treatment significantly decreased the expression levels of *Caspase 3* compared with the other groups, while miR-210-mimic treatment significantly increased *Caspase 3* expression levels compared with other groups (*p* < 0.05). Expression levels of *NANOG* and *SOX2* were significantly upregulated following the miR-210-inhibitor treatment compared with the other groups. In addition, miR-210-mimic treatment revealed that the expression levels of *NANOG* and *SOX2* were significantly different (*p* < 0.05) compared with the other groups.

## 4. Discussion

The presented study indicated that miR-210 affects the cleavage, blastocyst formation rate, and number of blastocyst cells. Our study revealed that microinjection of miR-210-inhibitor in porcine embryos significantly enhanced the cleavage rate, blastocyst formation rate, and TCN of blastocysts compared with the control and miR-210-mimic treatment groups. In contrast, the miR-210-mimic treatment negatively affected in vitro embryonic development as a result of reduced cleavage, blastocyst rate, and TCN of blastocysts compared with the other groups. Moreover, miR-210-inhibitor treatment resulted in significantly reduced expression levels of *XBP1* compared with control and miR-210-mimic, while miR-210-mimic treatment increased the expression levels of *XBP1* protein with significant differences compared with the other groups.

Studies of microRNAs were established in 1993 and have since developed to explain their structure, function, and role of action. Micro-RNAs are refined from the transcripts of RNA polymerase II/III. The miRNA genes are intragenic and can be transcribed independent of the host gene, using their own promoters. In addition, intragenic genes include the introns and exons of protein-coding genes [38,39]. Many reports have explained that miRNAs silence gene expression by suppressing translation and changing mRNA homeostasis [40,41,42]. In addition, miRNAs also play roles in several functions, including gene expression and transcriptional regulation [13,42,43]. Shoji and colleagues [13] reported that miR-210 is essential for cellular reaction to hypoxia, development of capillary-like structures, and VEGF-driven endothelial cell migration.

In this study, we applied an miR-210 inhibitor and mimic in parthenogenetically activated embryos in porcine embryos. We found that miR-210 inhibitor treatment significantly improved the cleavage rate, blastocyst rate, and TCN of blastocysts. The miR-210 mimic treatment resulted in a significant decrease in all parameters compared with the control and miR-210 treatments (*p* < 0.05). These results implied that inhibition of miR-210 supported in vitro embryo development, whereas treatment with the miR-210 mimic caused a decline in the development of porcine embryos during in vitro experiments. Studies related to the inhibition of miR-210 have been reported in various fields, including in brain injury in mice, hypoxic-ischemic encephalopathy (HIE) in rat animal models, neurotoxicity, and mitochondrial respiration in the placenta [17,28,44]. In line with previous reports, miR-210 treatment has many beneficial effects. A novel treatment using an miR-210 inhibitor significantly protected against acute ischemic brain injury in mice, and reduced cerebral infarct by alleviating pro-inflammatory cytokines such as interleukin 6 (*IL-6*), tumor necrosis factor (*TNF*)*-α*, *IL-1β*, and chemokines (*CCL2* and *CCL3*) [44]. Inhibition of miR-210 has also been shown to have a beneficial effect in the HIE of rats [17]. However, a study of the role of miR-210 in pre-eclampsia patients reported that increased expression levels of miR-210 resulted in mitochondrial dysfunction, increased ROS, and diminished oxygen absorption. Moreover, miR-210 inhibition protected mitochondrial function during respiratory insufficiency [28,29]. Therefore, it is clear that miR-210 is involved in various essential mechanisms, such as in vitro embryo development, cell metabolism, and the cellular stress response. Further, characterization of miR-210 has been reported in reproductive systems, and has been shown to be involved in cryptorchidism, spermatogenesis, and potentially in testis development [45]. Studies related to reproduction have further suggested that miR-210 has an essential function in placental development [39].

According to previous studies, inhibition of miR-210 by antagomir-210 resulted in a significant decrease in acanthosis and inflammatory cell infiltration; therefore, miR-210 inhibitor treatment has anti-inflammatory effects in mice [46]. MiR-210 also plays a role in adaptation to cellular stress [28,29]. During in vitro embryo culture, ER stress is reported as a major obstacle for in vitro embryo survival [47,48]. Accordingly, to understand the effects of miR-210 on ER stress, we analyzed the expression levels of *XBP1* in blastocysts using immunofluorescence staining. In the second experiment, the results revealed that the miR-210 inhibitor significantly decreased the fluorescence intensity compared with the control and miR-210 mimic. This result implied that an improvement in embryo development in vitro is correlated with a reduction in *XBP1* expression levels. In contrast, the miR-210 mimic showed significantly increased *XBP1* fluorescence intensity levels compared with control and miR-210 inhibitor treatment. The increase in *XBP1* expression levels occurred simultaneously with a decrease in cleavage rates during embryonic development and changes in the TCN of blastocysts. *XBP1* is commonly used as a marker of ER stress, both in vivo and in vitro [49,50]. In line with the present study, zinc treatment during IVM reported significantly reduced expression levels of *XBP1* in matured porcine oocytes; improved blastocyst formation rates; and significantly decreased gene expression of *Caspase 3* and ER stress-related genes such as *XBP1*, binding protein (*BiP*), *PTPN1*, and *ATF4* [51]. The increase of *XBP1* is suggestive of an activation of the ER stress response through the Inositol-requiring transmembrane kinase endoribonuclease-1α (*IRE1α*), one of the UPR branches. These mechanisms have been reported in all stages of mouse preimplantation [52]. Therefore, these results suggest the involvement of miR-210 in in vitro embryo development and TCN of blastocysts, and suggests a further detailed investigation linked with ER stress, apoptosis, and embryo quality.

In the third experiment, we analyzed the molecular aspects of UPR-related genes in porcine blastocysts [53], the apoptosis-related gene, *Caspase 3*, and the pluripotency-related genes *NANOG* and *SOX2*. To investigate ER stress signaling, we examined genes involved in the UPR mechanism. Microinjection of miR-210 inhibitor greatly reduced the expression of *uXBP1*, *sXBP1*, *ATF4*, and *PTPN1*. These results implied that miR-210 reduced ER stress through downregulation of UPR-related genes and resulted in improved development in porcine embryos. Furthermore, treatment with the miR-210 mimic significantly increased the expression of genes related to UPR signaling. Here, we showed that miR-210 mimics upregulated the expression of UPR-related genes (*uXBP1*, *sXBP1*, *ATF4*, and *PTPN1*) and impaired porcine in vitro embryo development by increasing ER stress-related gene expression. Thus, the development of in vitro embryos depends in part on the alleviation of ER stress through the unfolded protein response mechanism. In addition, the cellular demand for protein synthesis in the ER is balanced by its folding ability [53]. Previous studies reported that miR-210 targets (hypoxia-inducible factor) HIF-1α [54] and Prolyl 4-hydroxylase, beta polypeptide (*P4HB*) [32]. HIF-1α functions to trigger miR-210 expression and promote anti-proliferative and anti-apoptotic under hypoxic condition, and is regulated by VEGF [54,55]. The *P4HB* is a chaperone protein of ER stress signaling; it was reported that miR-210 was *P4HB*-targeting in temozolomide (TMZ)-resistance glioblastoma multiforme (GMB) cells [32]. The function of *P4HB* is to protect unfolded protein aggregation [56]. Therefore, miR-210 is associated with *P4HB* and ER stress. However, additional investigations are needed to uncover the mechanism of miR-210 in the ER stress pathway, particularly during in vitro embryo development.

In Brief, ER stress occurs as an imbalance between protein synthesis and secretion in the ER [52]. This condition can be triggered during the processes of in vitro embryo production, including oocyte retrieval, in vitro maturation, and manipulation of oocytes and embryos [52]. The mechanism underlying UPR activation for cell survival adaptation has been explained in a previous study [57]. Endoplasmic reticulum has three branch transmembrane proteins; dsRNA-activated protein kinase-like ER kinase (*PERK*), inositol-requiring enzyme 1 α (*IRE1α*), and activating transcription factor 6 (*ATF6*). These three branches were associated with glucose-regulated protein (*GRP*) *78* under normal conditions. Following an escalation in cellular stress conditions, *GRP78* is separated from the three transmembrane proteins, triggering UPR activation [49]. Separation of *GRP78* triggers the dimerization of PERK and the autophosphorylation of eukaryotic translation initiation factor 2 (e*IF2α*). Further, specific mRNAs targeting *ATF4* are translated to promote pro-survival and pro-apoptotic transcriptional processes [57]. During UPR activation, a branch of ER, *IRE1α* activates cytoplasmic kinase activity, phosphorylation, and endoribonuclease action [58]. Next, *IRE1α* endonuclease activity induces the conversion of *uXBP1* to *sXBP1*. Then, *sXBP1* activates UPR genes [49]. Upon *GRP78* separation from *ATF6*, *ATF6* translocates to the Golgi to produce soluble basic leucine zipper (bZIP). The combination of bZIP and ER stress response elements (ERSE-I and II) triggers ER stress response genes [59]. In addition, one study of cellular stress reported that ER stress induces the production of ROS and expression of *PTPN1*, an enzyme associated with ER stress, apoptosis, and steatosis [60]. Continuous ER stress may result in disrupted calcium homeostasis of ER, upregulating ROS levels, and ER impairment [61]. Calcium oscillations are essential during fertilized embryo development on pronucleus formation through regulation of the mitogen-activated protein kinase (MAPK) pathway [62]. In mice IVF embryo, the reduction of ER stress increased embryo development with normal neonatal weight [63]. Therefore, alleviation of ER stress also has a beneficial outcome on fertilized embryo development.

An examination of apoptosis-related genes showed that miR-210-inhibitor significantly reduced the expression of *Caspase 3*, while miR-210 has the opposite effect. Consistent with our findings, upregulation of miR-210 triggered pro-apoptotic expression of *Caspase 3* and upregulated endothelial cell apoptosis [29]. This finding implies that the miR-210-inhibitor also reduced apoptosis and improved in vitro embryo development. In contrast, the miR-210-mimic increased the apoptosis rate and decreased the cleavage rate, blastocyst rate, and TCN of blastocysts. In cases of severe ER stress, apoptotic cell death was induced by phosphorylated e*IF2α*, which further triggers *ATF4*, which induces apoptosis [60]. Our previous study showed that a reduction of ER stress-related genes and *Caspase 3* resulted in improved in vitro embryo development [51]. These results revealed that miR-210 is involved in alleviating apoptosis by decreasing *ATF4* and *Caspase 3*. Investigation of embryo quality-related genes revealed that the expression levels of *NANOG* and *SOX2* were significantly improved in blastocysts derived from the miR-210-inhbitor cells. Nevertheless, treatment with an miR-210-mimic significantly decreased the expression levels of *NANOG* and *SOX2* compared with the control and miR-210 treatment. In line with this, miR-210 overexpression was shown to decrease *P4HB*, a chaperone protein related to the ER stress response, in glioblastoma multiforme cells study [32]. Experiments in buffalo rat liver cells (normal rat hepatocytes) revealed that the miR-210 inhibitor stimulated cell proliferation; however, the miR-210 mimic suppressed cell proliferation at 16, 20, and 24 h in vitro [64]. One study in mice revealed that an miR-210 mimic disrupted mitotic progression and resulted in abnormal mitosis [65]. Thus, miR-210 mimic could impair the ER stress response, disturb the cellular microenvironment, and affect embryo quality by regulating *Caspase 3* and pluripotency-related genes (*NANOG* and *SOX2*). The miR-210 mimic is designed to be 21 base pairs, double-stranded RNA (dsRNA) oligonucleotides. In addition, to avoid non-specific impact caused from the cellular dsRNA-dependent protein kinase (PKR) response, small interfering RNAs (siRNAs) are designed to be <~30 base pairs for mammalian cells [66]. Moreover, non-specific cellular responses are dependent upon the concentration of siRNA [67]. The dsRNAs bind and trigger PKR, afterwards stimulating non-specific mRNA degradation and apoptosis [68]. Therefore, the non-specific effect should be considered in the siRNA experiment and further study [67]. Regarding the treatment of miR-210 inhibition and mimic, further studies such as the possible side effects on the embryo and mechanisms of cellular response under miR-210 treatment on embryo are needed for further investigation. 

## 5. Conclusions

Altogether, we demonstrated that miR-210 treatment plays an essential role in the development of porcine embryos. This is evident in the observed improvement of cleavage, blastocyst rate, and TCN of blastocysts when treated with miR-210-inhibitor. In contrast, treatment with the miR-210-mimic resulted in lower in vitro embryo development and TCN of blastocysts. In a subsequent analysis, the miR-210-inhibitor reduced the fluorescence intensity levels of *XBP1* compared with the control and miR-210-mimic in cleavage and blastocyst stage. Furthermore, miR-210-inhibitor also alleviated ER stress and apoptosis by downregulating the expression levels of *uXBP1*, *sXBP1*, *ATF4*, *PTPN1*, and *Caspase 3*. In addition, a significant improvement in the gene expression levels of pluripotency-related genes (*NANOG* and *SOX2*) was achieved with miR-210-inhibitor treatment. Therefore, we suggest that miR-210-inhibitor treatment alleviates ER stress and reduces apoptosis to support the enhancement of in vitro embryo production. However, further investigation of miR-210-related ER stress in preimplantation embryos is needed to understand the pathway and mechanism of miR-210 in porcine embryos.

## Figures and Tables

**Table 1:** Micro-RNA-210 sequence for microinjection.

Product Number	Micro-RNA	Sequences (5′-3′)	Base Count
oligo-rna-single-customorder	cfa-miR-210 inhibitor	UCAGCCGCUGUCACACGCACAGU	23
oligo-rna-double-customorder	cfa-miR-210 mimic	S-ACUGUGCGUGUGACAGCGGCUGA AS-UCAGCCGCUGUCACACGCACAGU	21

S, sense; AS, antisense.

**Table 2:** Primer sequences for real-time PCR.

Genes	Primer Sequences (5′-3′)	Product Size (bp)	Accession No.
*GAPDH*	F: GTCGGTTGTGGATCTGACCT R: TTGACGAAGTGGTCGTTGAG	207	NM_001206359
*ATF4*	F: AGTCCTTTTCTGCGAGTGGG R: CTGCTGCCTCTAATACGCCA	80	NM_001123078.1
*PTPN1/* *PTP1B*	F: GGTGCTCACGACTCTTCCTC R: TTCTCTGCACGAGCTTCTGA	158	NM_001113435.1
*uXBP1*	F: CATGGATTCTGACGGTGTTG R: GTCTGGGGAAGGACATCTGA	106	NM_001142836.1
*sXBP1*	F: GGAGTTAAGACAGCGCTTGG R: GAGATGTTCTGGAGGGGTGA	142	NM_001271738.1
*Caspase 3*	F: GCCATGGTGAAGAAGGAAAA R: GGCAGGCCTGAATTATGAAA	132	NM_214131.1
*NANOG*	F: GGTTTATGGGCCTGAAGAAA R: GATCCATGGAGGAAGGAAGA	98	NM_001129971
*SOX2*	F: ATGCACAACTCGGAGATCAG R: TATAATCCGGGTGCTCCTTC	130	NM_001123197

PCR, polymerase chain reaction; F, forward primer; R, reverse primer.

**Table 3:** Effects of miR-210 (inhibitor and mimic) treatment on porcine in vitro embryo development.

Treatment	Number of Embryos Cultured	No. of Embryos Developed to (Mean ± SEM, %)		TCN (Mean ± SEM)
		≥2 cells	Blastocyst	
Control	223	193 (86.61 ± 0.52) ^a^	46 (20.61 ± 0.57) ^a^	65.58 ± 0.95 ^a^
miR-210-inhibitor	225	212 (90.25 ± 0.5) ^b^	79 (33.19 ± 2.37) ^b^	76.25 ± 1.11 ^b^
miR-210-mimic	235	165 (73.28 ± 1.4) ^c^	33 (14.6 ± 0.46) ^c^	49.33 ± 0.76 ^c^

Replication number = 6. SEM, standard error of mean; TCN, total cell number. ^a–c^ within a column, value with different superscript letters are significantly different (*p* < 0.05).

## Data Availability

Data supporting the results of this study shall, upon appropriate request, be available from the corresponding author.
